# The application of hand‐foot‐combined‐usage in learning the structure of epidermis

**DOI:** 10.1111/srt.13094

**Published:** 2021-08-22

**Authors:** Yue He, Yan Duan, Ruiya Li, Jian Wang

**Affiliations:** ^1^ Inner Mongolia Medical University Department of Graduate School Inner Mongolia China; ^2^ Department of Dermatology and Venereology Inner Mongolia People's Hospital Inner Mongolia China

Dear Editor,

Skin, the largest organ of the human body, is organized into an elaborate layered structure consisting mainly of the outermost epidermis and the underlying dermis. Teaching dermatology to medical students entails a series of lectures, pictures, and examinations to convey a sense of skin features and textures, often by use of simulated skin models, it is urgent to incorporate newer teaching methods to explain the structure of epidermis.

“Hand‐foot‐combined‐usage” method is a new teaching mode, and we read an article “Teaching application of hand‐foot‐combined‐usage in supracondylar fracture of femur” published by Li et al.^1^ in your journal. Therefore, we are inspired to represent the structure of the epidermis.

We can use “hand‐foot‐combined‐usage” to demonstrate the delamination of the epidermis (Figures 1 and 2), so as to interpret the layering of the epidermis. The five fingers of the hand from the small finger to the thumb represent stratum basale, stratum spinosum, stratum granulosum, stratum lucidum, and stratum corneum. Stratum basale: It consists of a cylindrical layer of basal cells, usually arranged in a palisade shape. The nucleus is oval and the nucleolus is obvious. Stratum spinosum: This layer consists of four to eight layers of polygonal cells. Stratum granulosum: It is usually composed of one to three layers of flat or rhomboid cells. Stratum lucidum: It is located between stratum granulosum and stratum corneum, and is only found in the thicker epidermis such as palm and plantar. Stratum corneum: It is located in the top layer of epidermis, and this layer of cells has no nucleus.

**FIGURE 1:**
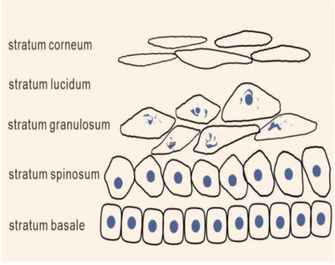
The epidermis

**FIGURE 2:**
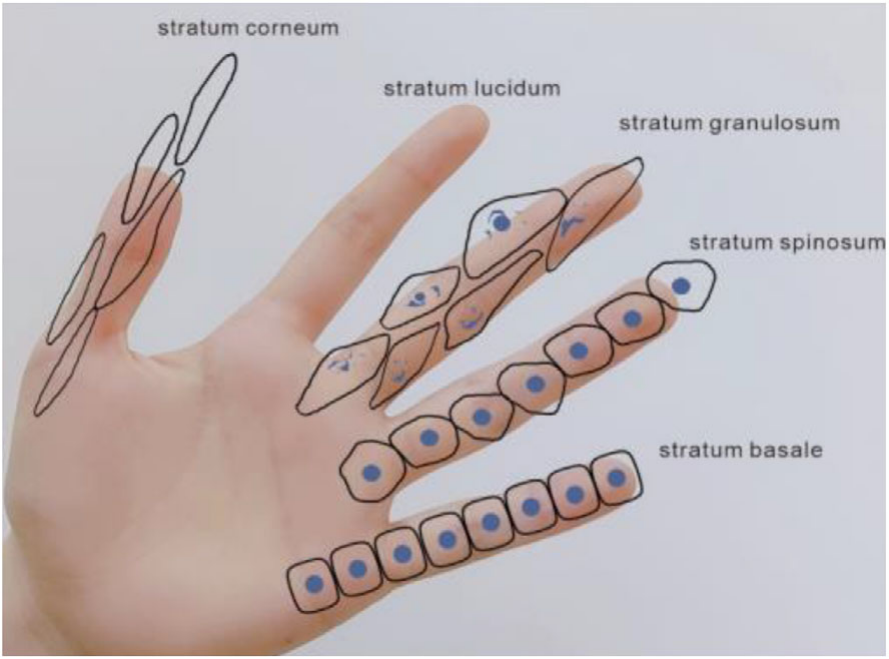
Simulate the epidermis by hand

We can also use the method to demonstrate the evolution of keratinocytes in the epidermis (Figure 3). The evolution of keratinocytes can generally be divided into four layers, because the stratum lucidum is rare. Epidermal renewal time refers to the time when cells proliferate and differentiate from basal layer to stratum spinosum and further differentiate into inactive stratum corneum, which is usually 52–75 days, but in some diseases such as psoriasis it will be greatly shortened.

**FIGURE 3:**
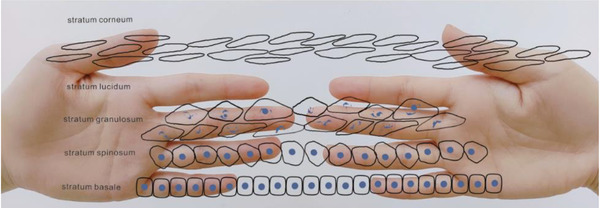
Simulate the evolution of keratinocytes in the epidermis by hand

By using this method to explain the process of keratinocytes, we can understand the occurrence, development, and outcome of related diseases. We can show the thickening of a certain layer by adding fingers (Figures 4, 5, 6). For example, hyperkeratosis (Figure 4) is caused by an abnormal thickening of the stratum corneum caused by increased deposition of keratin which can be seen in lichen planus, palmoplantar keratosis, ichthyosis, etc. Hypergranulosis (Figure 5) refers to the increase of stratum granulosum cells, which are found in lichen simplex chronicus, lichen planus, etc. Acanthosis (Figure 6) indicates an increase in the number of cells in the stratum spinosum, and is found in chronic dermatitis and psoriasis.

**FIGURE 4:**
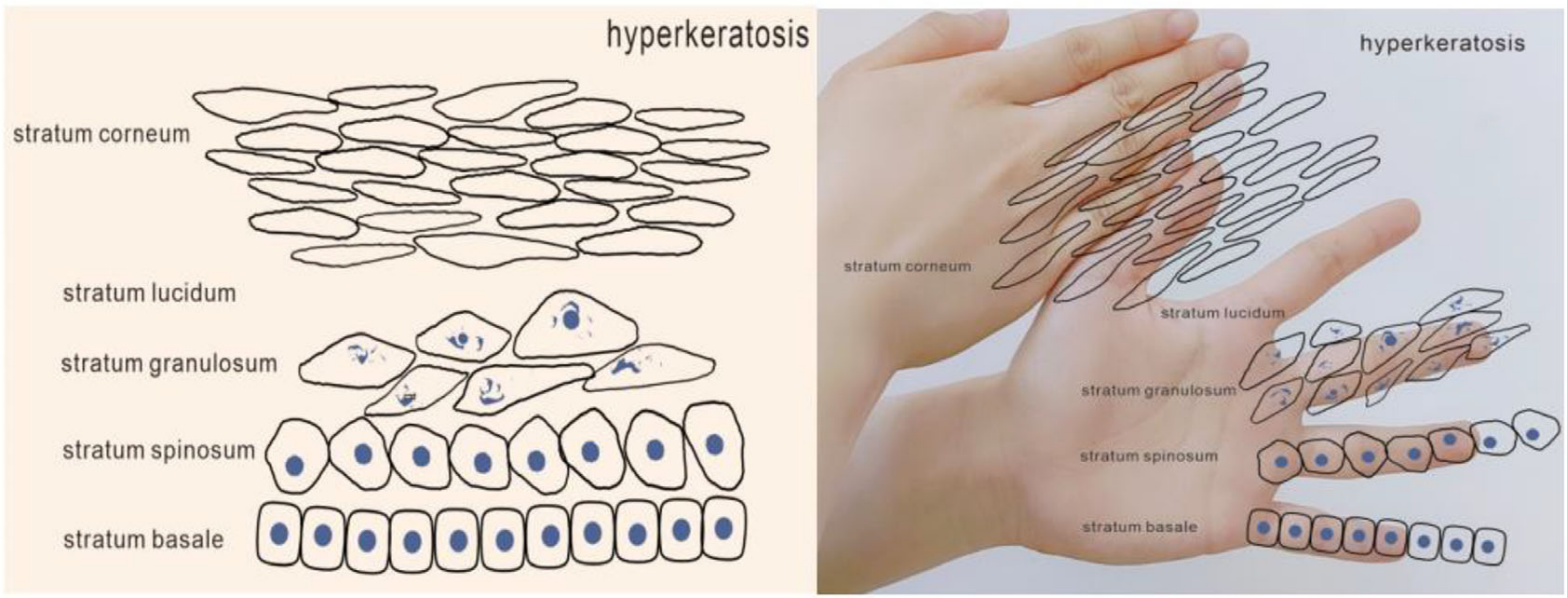
Simulate the hyperkeratosis by hand

**FIGURE 5:**
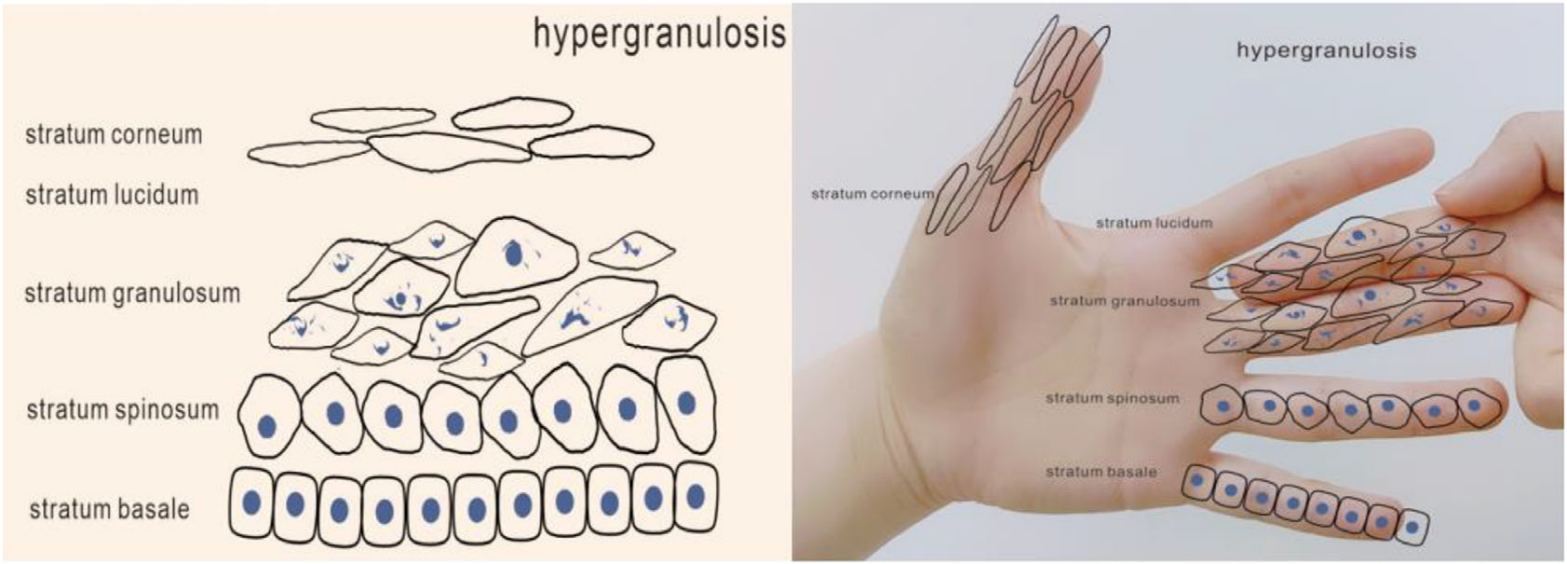
Simulate the hypergranulosis by hand

**FIGURE 6:**
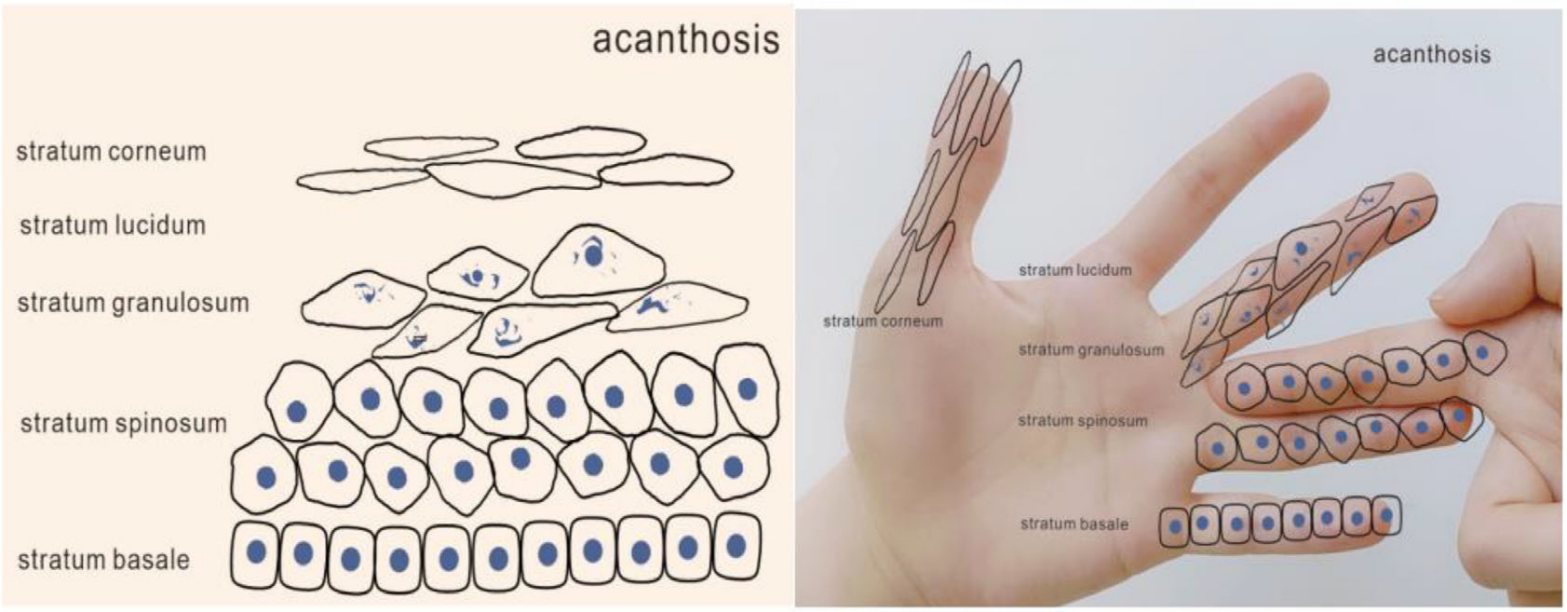
Simulate the acanthosis by hand

Although “hand‐foot‐combined‐usage” is novel compared to other teaching methods, its method is similar to skin structure. This new teaching helps students have a clear understanding of the structure and evolution of the epidermis, which will facilitate easy understanding of skin pathologies and diseases.

## CONFLICT OF INTEREST

All authors have no conflict of interest.
